# Resumé of Our Actual Knowledge of the Relation of the Organs of Vision to the Nervous Centers

**Published:** 1885-10

**Authors:** 


**Riassunto delle Attuali Nostre Cognizioni sui Rapporti dell'Apparecchio Visivo coi Centri Nervosi.** (Resumé of our Actual Knowledge of the Relation of the Organs of Vision to the Nervous Centers.) By Prof. ANTONIO QUAGLINO, of Pavia.

These two pamphlets will be of great interest and value not only to the oculist but also to the physiologist and neurologist. In the brief space of sixty octavo pages, have been recorded all discoveries and the results of all experiments made in connection with these subjects from the time of Gian Porta—who, in 1400, compared the eye to a camera obscura—to the present day. These articles are well worthy of translation.

